# The Influence of Self-Myofascial Release on Muscle Flexibility in Long-Distance Runners

**DOI:** 10.3390/ijerph19010457

**Published:** 2022-01-01

**Authors:** Iwona Sulowska-Daszyk, Agnieszka Skiba

**Affiliations:** Institute of Clinical Rehabilitation, University of Physical Education in Krakow, 31-571 Krakow, Poland; agnieszka.skiba@awf.krakow.pl

**Keywords:** self-myofascial release, foam rolling, muscle flexibility, foam roller

## Abstract

During long-distance running, athletes are exposed to repetitive loads. Myofascial structures are liable to long-term work, which may cause cumulating tension within them. The aim of this study was to evaluate the acute effect of self-myofascial release on muscle flexibility in long-distance runners. The study comprised 62 long-distance, recreationally running participants between the age of 20 and 45 years. The runners were randomly divided into two groups: Group 1 (*n* = 32), in which subjects applied the self-myofascial release technique between baseline and the second measurement of muscle flexibility, and Group 2 (*n* = 30), without any intervention. The self-myofascial release technique was performed according to standardized foam rolling. Assessment of muscle flexibility was conducted according to Chaitow’s proposal. After application of the self-myofascial release technique, higher values were noted for the measurements of the following muscles: piriformis, tensor fasciae latae muscles and adductor muscles. Within the iliopsoas and rectus femoris muscles, lower values were observed in the second measurement. These changes were statistically significant (*p* < 0.05) within the majority of muscles. All these outcomes indicate improvement related to larger muscle flexibility and also, an increase in range of motion. In the control group (Group 2), significant improvement was observed only in measurements for the iliopsoas muscles. The single application of self-myofascial release techniques with foam rollers may significantly improve muscle flexibility in long-distance runners. Based on these results, the authors recommend the self-myofascial release technique with foam rollers be incorporated in the daily training routine of long-distance runners, as well as athletes of other sport disciplines.

## 1. Introduction

Running is a natural form of movement and one of the most popular types of activity. However, as in the case of other sport disciplines, running involves being subjected to risk of injuries and overloads. With the increase in popularity of this form of physical activity, the frequency of injuries associated with its practice also increases, especially within the lower limbs [1,2,3]. The highest activity of the lover limb muscles occurs immediately before initial contact and at the beginning of the support phase. The increased range of movement in the joint during running elongates the time of muscle activity. During long-distance running, athletes are exposed to repetitive loads. Myofascial structures are liable to long-term work, which may cause cumulating tension within them. This is especially true in the case of eccentric work of the hamstring during the terminal swing phase, which may lead to excessive tension and reduction of this flexibility within this muscle group. Thus, maintaining proper muscle flexibility is crucial for long-distance runners [4].

Flexibility may be defined as the property and ability of body tissues to achieve full range of motion (ROM) without any injury to the joints or within their groups. Range of motion is regulated by proper extensibility of all soft tissues encompassing the joints [5,6]. The basic role of flexibility is to reduce the risk of injury. Proper muscle elasticity increases the ability to move joints within their maximal possible range of motion. Moreover, flexibility exercises or techniques used before a main training event may enhance physical performance, especially that of muscle strength. This is achieved by increasing the use of elastic strain energy during the performance of movements [7]. One technique, the aim of which is to increase flexibility of the soft tissues, is myofascial release (MFR). MFR is based on manual therapy and helps reduce restrictions or adhesions within layers of the fascial tissue [8]. Myofascial release includes different procedures, such as structural integration (Rolfing), osteopathic soft-tissue techniques, massage, trigger point release, the muscle energy technique and others [9]. Most of them are passive techniques in which the patient is dependent on a therapist [10].

A special technique within MFR is self-myofascial release (SMFR). In contrast to those techniques mentioned above, SMFR is performed by the patient independently, instead of by a therapist. This technique utilizes the patient’s body mass and special tools such as massage balls or foam rollers to apply pressure and stretch the restricted soft tissue [10,11,12]. Foam rollers used in the SMFR technique are cylinders made of foam with various texture, size and density [10,13]. Foam rollers have their usage in treating large groups of muscles with a specified protocol of starting and ending position [14]. SMFR involves movements back and forth over the tool, from the proximal to distal part of the muscle groups, and inversely. In the case of the myofascial trigger points (MTrP), the SMFR technique is concentrated over the painful area to provide sustained compression on the MTrP [10,15]. The effectiveness of SMFR is explained by sweeping and direct pressure on soft tissue, which may cause warming of the fascia, ripping of fibrous adhesions and restrictions within layers of the fascia and restoring soft tissue elasticity [16].

Moreover, fascial elasticity is contingent upon tissue hydration. The more hydrated the tissues, the less rigid they are. Some parts, where the fascia is less elastic because of fibrous adhesions and restrictions, are less hydrated. The SMFR technique allows to increase elasticity of the fascia and the degree of its hydration. During compression via foam rollers, the fascia has been shown to extrude water. After rolling, when pressure on the fascia decreases, there is a re-inflow of water from the surrounding tissues, as well as the lymphatic and vascular networks. Compression during the SMFR technique may increase fascial elasticity and compliance through a temporary change in water content [17,18]. Another explanation indicates ischemic compression during performance of the SMFR technique. The local blood flow is increased after achieving compression. As a result, removal of metabolites, delivering oxygen and tissue treatment are facilitated [19,20,21].

There are many studies in which the effectiveness of SMFR using foam rollers is shown. This technique is commonly recommended as a part of the warm-up phase due to its positive influence on the length-to-tone ratio within the muscles [22]. It can be both a form of warm-up or cool-down of the body after physical activity [14]. It may also be used in the prevention of injuries. SMFR is a technique applied to restore proper tension of tissues, increasing their flexibility [16,23,24,25], removing trigger points [8,26] and enhancing muscle recovery after exercise [14,27,28].

However, despite the many existing studies on this topic, none of them involves the influence of SMFR on muscle flexibility in runners. The flexibility and proper functioning of myofascial chains are crucial for athletes. Due to myofascial structure continuity, overloading forces may be transferred by the myofascial system, leading to tissue overload, repetitive strain injuries, resulting restrictions in muscle flexibility and disruptions in functional movement patterns. Moreover, restrictions in one part of the body may cause excessive tension in others [29]. There are some studies in which a concern is put forward, stating that chronic endurance training may induce decreased flexibility [30,31]. An optimal level of this parameter is necessary for health. Higher muscle flexibility can produce a protective role against muscle damage during exercise [32,33]. Long-distance running is one of the most popular sport disciplines wherein athletes are exposed to repetitive loads; thus, improving flexibility should be crucial in this discipline. In the current study, this issue is undertaken for the first time. The aim of this study was to evaluate the acute effect of self-myofascial release on muscle flexibility in long-distance runners.

## 2. Materials and Methods

### 2.1. Study Group

The study comprised 62 long-distance, recreationally running participants (18 females and 44 males), aged 20–45 years (mean ± SD 33.79 ± 7.56). The participants ran regularly at a total distance of 30–100 km per week (mean ± SD 48.15 km ± 17.02 km). The recruitment of runners was performed according to exclusion and inclusion criteria. The inclusion criteria were as follows: weekly covered distance of 30 km or more, regular running training, age between 20 and 45 years, no deformation of the feet, no acute injury lasting up to 6 months prior to enrolment in the study and consent for participation. The inclusion criteria were created on the basis of data from the International Institute for Race Medicine [34].

The exclusion criteria were as follows: weekly distance of less than 30 km, irregular running training, age above 45 or less than 20 years, visible deformation of the feet, previous history of acute injury lasting up to 6 months prior to enrolment in the study, chronic pain, systemic disease (e.g., hypertension, diabetes, fibromyalgia) or lack of consent to participate in the study. The subjects were requested to refrain from physical activity 24 h prior to measurements and foam rolling 3 days before testing.

Before the study, all of the participants were informed about the research procedures as well as the purpose of the study in detail and provided their written informed consent to participate in the research. This study was registered in the Australian New Zealand Clinical Trials Registry (ANZCTR) and the written approval of The Ethical Committee of Regional Medical Chamber was obtained (No. 40/KBL/OIL/2015). All the procedures complied with the 1964 Declaration of Helsinki.

The runners were randomly divided into 2 groups: Group 1 (*n* = 32), in which subjects applied the self-myofascial release technique between baseline and the second measurement of muscle flexibility, and Group 2 (*n* = 30), without any intervention. The researchers used simple randomization by flipping a coin. The researcher was blinded to the subject group allocation. A detailed characterization of both groups is presented in Table 1.

### 2.2. Procedures

Before the testing session, participants from Group 1 were familiarized with the proper performance of self-myofascial release using foam rollers. Three days after familiarization, the runners were invited for the proper testing session.

The testing session began with a 5 min warm-up during which participants of both groups ran at a comfortable pace. Then, muscle flexibility was assessed twice: at baseline and after 15 min. Following the first examination, each participant from Group 1 applied the self-myofascial release technique using foam rollers on the following muscles: hamstring, gluteus maximus, hip adductors, quadriceps, tensor fasciae latae and gastrocnemius. Runners from Group 2 did not perform any of the techniques between first and second measurements.

In that study, a 45cm high and 14cm diameter, high-density 4Fizjo brand foam roller was used.

SMFR using foam rollers was performed according to the standardized foam rolling procedure [35]. This technique was applied along the muscle fibers, from proximal to distal muscle insertion, and inversely, with a constant pressure and speed of 2.5 cm/s. The correct speed of SMFR was demonstrated and then monitored by the researcher while the subject performed the technique. Participants repeated this technique 10 times for each muscle group. The SMFR was applied an average of 2 min for each muscle group. Foam rolling was performed on both lower limbs. The SMFR was applied only on the muscle tissue, avoiding pressure on bones, joints or tendons.

#### 2.2.1. Rolling of the Hamstring

**Figure 1:**
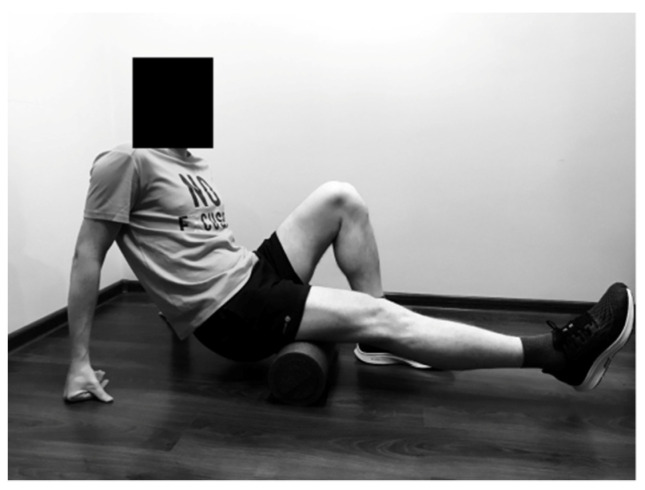
Rolling of the hamstring.

The SMFR technique was performed in back support position with hands on the floor. The runner rolled 1 lower limb, starting above the popliteal fossa towards the ischial tuberosity, and inversely. The second leg was braced on the ground as support (Figure 1).

#### 2.2.2. Rolling of the Gastrocnemius

**Figure 2:**
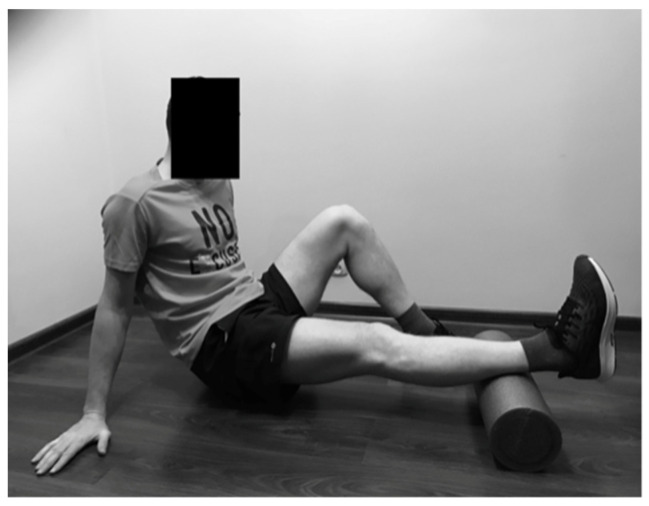
Rolling of the gastrocnemius.

The SMFR technique was performed in back support position with hands on the floor. The study participant put the foam roller under the calf, with the knee joint in extension. The foot of the second lower limb was supported on the ground. The subject moved back and forth over the foam roller starting over the Achilles tendon towards the knee joint, and inversely (Figure 2).

#### 2.2.3. Rolling of the Gluteus Maximus

**Figure 3:**
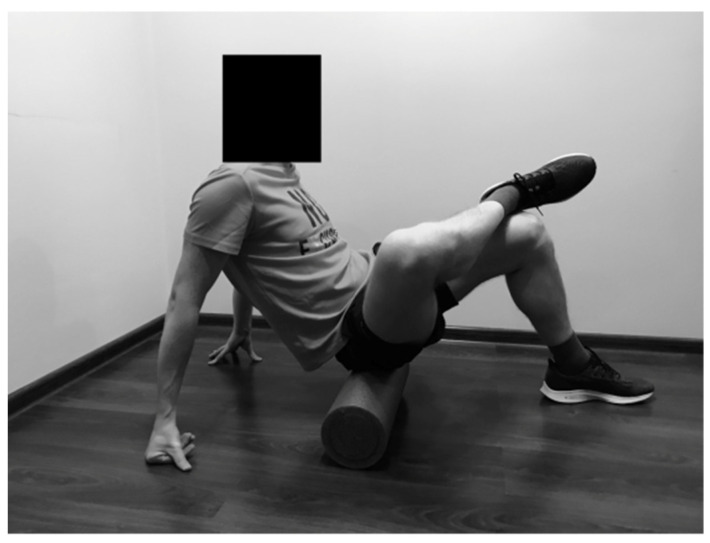
Rolling of the gluteus maximus.

The study participant sat on the foam roller, crossing 1 foot over the opposite knee. The hands were placed on the floor behind the back. The runner moved back and forth over the foam roller to roll the gluteus muscle (Figure 3).

#### 2.2.4. Rolling of the Hip Adductors

**Figure 4:**
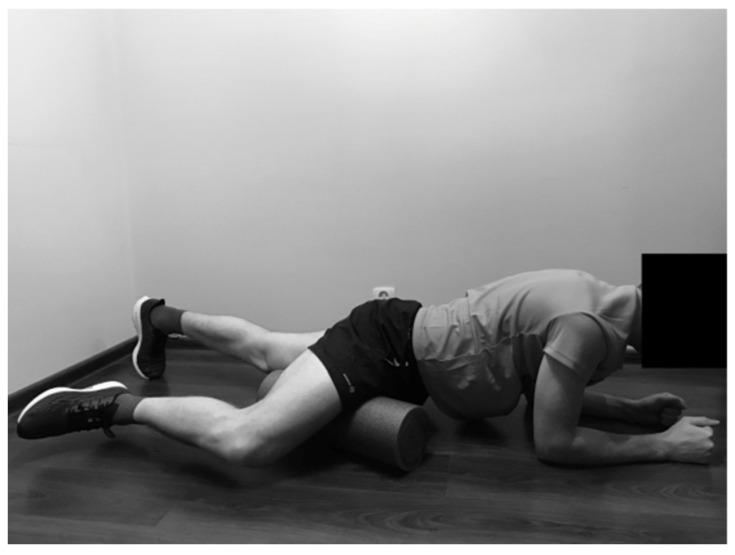
Rolling of the hip adductors.

The SMFR technique was applied in front support position on the forearms. One lower limb was placed on the foam roller in flexion, with external rotation and abduction in the hip joint. The subject moved back and forth over the foam roller, starting over the knee joint towards the groin, and inversely (Figure 4).

#### 2.2.5. Rolling of the Quadriceps

**Figure 5:**
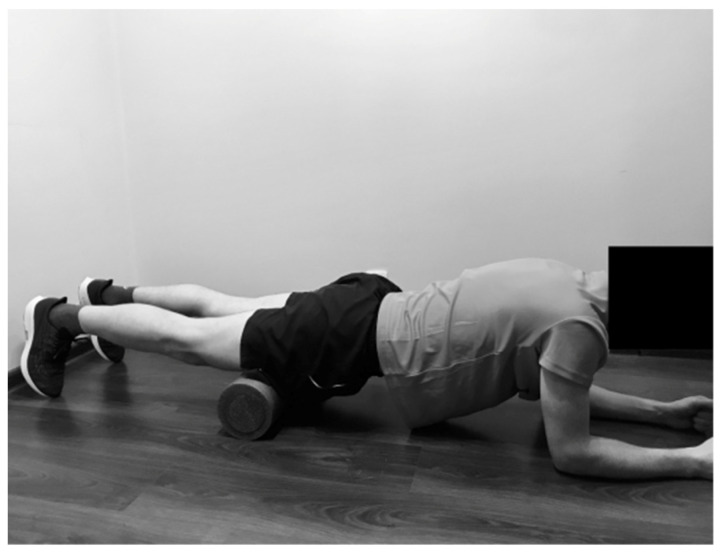
Rolling of the quadriceps.

The SMFR technique was performed in front support position on the forearms. Both lower limbs were placed on the foam roller. The subject moved back and forth over the foam roller, starting over the knee joint towards pelvis, and inversely (Figure 5).

#### 2.2.6. Rolling of the Tensor Fasciae Latae

**Figure 6:**
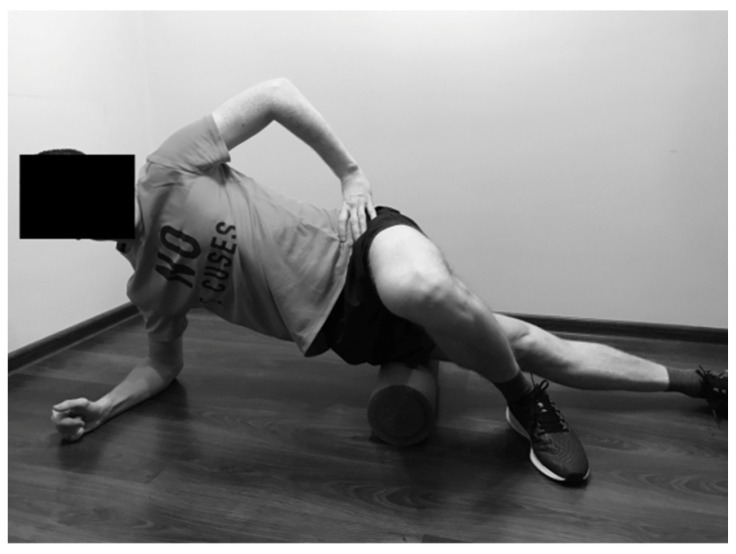
Rolling of the tensor fasciae latae.

The runner was in side-bridge position with the thigh of the lower leg placed on the foam roller. The top leg was crossed over the lower leg, with the foot supported on the ground. The SMFR technique was performed from the hip joint towards the knee joint, and inversely (Figure 6).

### 2.3. Research Tool

Data were collected at the research laboratory. Assessment of muscle flexibility was conducted according to Chaitow [36] to the nearest 0.5 cm. For measurements, a centimeter tape was used. The following muscles were assessed:

The iliopsoas muscle was tested with the modified Thomas test. The subject sat at the edge of a couch, then rolled back onto the coach while pulling both knee joints to the chest. The subject held the opposite hip joint in maximal flexion with the arms, while the lower limb to be tested was lowered towards the floor. The distance between the table and the midpoint on the patella lateral edge was measured.

The rectus femoris muscle was evaluated during prone position, in a relaxed state. The researcher stood next to the participant, at the side of the lower extremity being tested. One hand of researcher was placed on the lower back while the other held the leg at the heel. The knee joint was passively flexed. The distance between the table and the lateral malleolus was measured.

The adductor muscles were assessed in supine position of the subject, with maximal adduction in the hip and knee joints, which were extended. The distance between the left and right femoral medial epicondyles was measured.

The tensor fasciae latae muscle was tested in side-lying position. The bottom knee and hip joints were flexed to flatten the lumbar curvature. The researcher stood behind the subject and stabilized the pelvis. The distal end of the evaluated lower extremity was held with the researcher’s other hand. The evaluated limb was lowered towards the floor with maximal external rotation, extension and adduction of the hip joint. The distance between the table and lateral malleolus was measured.

The external rotation muscles (piriformis muscles) were evaluated with the subject in prone position. The knee joints were flexed to a 90° angle, and maximal internal rotation of the hip joints was performed. The distance between the left and right medial malleoli was measured.

The quadratus lumborum muscle was tested in standing position. The displacement in finger position between standing in a relaxed position and the maximal side flexion of the trunk was measured.

### 2.4. Statistical Analysis

Statistical analysis was performed using STATISTICA 12.0 Pl software (StatSoftPolska, Krakow, Poland). The data obtained in the study were presented in the form of mean values and standard deviations. To assess data for normality, the Shapiro–Wilk test was conducted. To determine the significance of the differences of muscle flexibility measurements, two-way ANOVA was performed with 1 main factor being between subjects (Group 1 and Group 2), and the other main factor being a repeated measure (time: baseline and after). Posthoc analysis was carried out using the Tukey’s posthoc test. The differences were considered statistically significant if the level of test probability was lower than the assumed level of significance (*p* < 0.05). Using the paired *t*-test for power analysis of exercise, it was determined that at least 30 subjects from each group were required to obtain a power of 0.8 at the two-sided level of 0.05, with the effect size of d = 0.8.

## 3. Results

After application of the self-myofascial release technique using foam rollers, higher values were noted in the measurement of the following muscles: piriformis (external rotation muscles), tensor fasciae latae muscles and adductor muscles. Within the iliopsoas and rectus femoris muscles, lower values were observed in the second measurement. These changes were statistically significant in the measurement of the following muscles: iliopsoas left and right, tensor fasciae latae left and right, rectus femoris left and right. In Group 2, a significant change was observed only for the measurement of the iliopsoas muscles (Table 2).

## 4. Discussion

The aim of this study was to evaluate the acute effect of self-myofascial release on muscle flexibility in long-distance runners. The most novel finding of this study is that single use of the self-myofascial release technique with foam rollers may significantly improve muscle flexibility in these subjects. In the experimental group (Group 1), after application of the SMFR technique in the piriformis (external rotation muscles), adductor and tensor fasciae latae muscles, higher values were noted, whereas lower values were observed in the rectus femoris and iliopsoas muscles. All these outcomes indicate improvement related to larger muscle flexibility, and following, an increase in range of motion. In the control group (Group 2), significant improvement was observed only in measurements for the iliopsoas muscles.

There are no similar studies focused on the influence of the self-myofascial release technique with foam rollers within the lower limb muscles on muscle flexibility in long-distance runners. In the literature, there is some research in which the influence is described with regard to myofascial release techniques in other groups of athletes or patients.

One of the studies investigating the impact of self-massage with a roller massager was performed by Halperin et al. [24]. The aim of that study was to compare the effects of static stretching and self-massage with roller massage of the calf muscles. The researchers measured maximal voluntary contraction, force, ankle ROM, single limb balance and electromyographic characteristics of the plantar flexors. The study participants included 14 recreationally trained subjects, who performed physical activity at least 2 days a week. Both roller massage and static stretching significantly improved ankle ROM to a similar degree. Moreover, the self-massage technique increased force production. In their conclusions, the authors indicated the possibility of using that technique as part of the warm-up. Similar to Halperin et al. [24], in the authors’ study, the participants used foam rollers to perform the self-myofascial release technique. However, in our study, SMFR was applied on several muscle groups of the lower limbs. The obtained results are consistent with the cited research and also indicate a positive influence of the applied technique on muscle flexibility.

Skarabot et al. [37] evaluated the effects of static stretching, foam rolling and a combination of both on passive ROM ankle dorsiflexion. The above techniques were applied within the plantar flexor muscles among resistance-trained athletes. The authors observed that all of these techniques caused enhanced flexibility, but a combination of static stretching and foam rolling seemed to have an additive effect in comparison to foam rolling alone [37]. Comparison of foam rolling and static stretching was performed by Mohr et al. [38]. These researchers concluded that using the foam roller for 3 sets of 2 min repetitions may increase hip flexion ROM. Nevertheless, the most significant effects were obtained using foam rollers in combination with a static-stretching protocol [38]. In the current study we have not evaluated other techniques. The use of only the self-myofascial release technique showed improvement in flexibility of the assessed muscle groups.

In a study conducted by MacDonald et al. [16] the aim was to determine the effect of the self-myofascial release technique using foam rollers on knee joint ROM and knee extensor muscle activation as well as force. The authors reported that foam rolling applied on the quadriceps femoris muscles significantly increased range of motion in the knee joints. The same technique used for the hamstrings increased range of motion in the Sit-and-Reach test and improved the flexibility of these muscles [39,40]. The results obtained in the present work are consistent with cited studies. Admittedly, in our research, we have not evaluated muscle flexibility of the hamstring, but we have observed significant improvement of rectus femoris flexibility.

On the other hand, there are some studies in which self-myofascial release is described as a technique without influence on range of motion. According to the research by Morton et al. [41], the obtained results may suggest that the addition of self-myofascial release techniques to static stretching does not enhance the efficacy of static stretching alone. In the above article, the authors assessed the SMFR technique applied only on the hamstring. Perhaps, if the researchers applied the technique to other muscles of the superficial back line, opposite results would be obtained. Peacock et al. [42] evaluated the influence of foam rolling on performance in addition to a dynamic warm-up. Flexibility was measured by the Sit-and-Reach test. The results obtained by the authors suggest that a warm-up with foam rolling exercises was unsuccessful for the improvement of flexibility, but it has the potential to improve test results for power, speed and agility performance [42].

The authors’ study was focused on the acute influence of the self-myofascial release technique, in comparison to measurements without any intervention. The results suggest that the single use of the self-myofascial release technique with foam rollers may significantly improve muscle flexibility in long-distance runners. In the experimental group, the obtained results indicate improvement related to larger muscles flexibility and a following increase in range of motion. In the control group, significant improvement was observed only in measurements of the iliopsoas muscles. The preferable outcome obtained in the second measurement of this muscle may result from the fact that the result of the modified Thomas test was positive in the assessment of each runner. According to a previous study [43,44,45,46], inability of the thigh to extend to neutral or drop below the horizontal position represents a positive test, which indicates restriction of muscle flexibility. Perhaps in the case of elasticity restrictiveness, the first measurement may have been enough stimulus to increase its flexibility.

There are some limitations of this study that should be addressed. First of all, the total running distance of the subjects ranged between 30 and 100 km per week, which resulted in some heterogeneity of the group. Moreover, the number of males and females differed. The next study limitation was the lack of blindness of examiners. In the current study, the authors investigated single application of the self-myofascial release technique with foam rollers. Moreover, the improvement in flexibility was evident within the group, but there were no statistically significant differences between groups. In further studies, it is recommended to assess the long-term use of this technique. Finally, it would also be worth assessing the impact of SMFR on the potential risk of injury in long-distance runners.

The outcomes obtained in the authors’ study allow to suggest that single application of the self-myofascial release technique with foam rollers may significantly enhance muscle flexibility in long-distance runners. Muscle flexibility and proper functioning of myofascial chains are extremely important for athletes, especially in the case of long-distance runners who are liable to repetitive and long-term loadings. Restrictions in one part of the body may cause excessive tension in others according to the myofascial structure’s continuity [29]. The overloading forces may also be transferred by the myofascial system, leading to tissue overload, repetitive strain injuries and resulting restrictions in muscle flexibility [26]. Due to increased muscle flexibility after applying the SMFR technique, this may potentially decrease the risk of injuries and overloads. Based on the results obtained in this study, the authors recommend the self-myofascial release technique with foam rollers be incorporated in the daily training routine of long-distance runners, as well as athletes of other sport disciplines.

## 5. Conclusions

Single application of the self-myofascial release techniques with foam rollers may significantly improve muscle flexibility in long-distance runners. Including the SMFR in the daily training routine of long-distance runners, as well as athletes representing other sport disciplines, is recommended.

## Figures and Tables

**Table 1:** Detailed characteristics of the groups.

	Group 1 (*n* = 32) Mean ± SD	Group 2 (*n* = 30) Mean ± SD
Age	34.09 ± 7.73	33.46 ± 7.33
Males	22	18
Females	10	12
High [cm]	175.81 ± 8.73	177.60 ± 7.63
Body mass [kg]	69.88 ± 9.55	70.70 ± 8.79
Total distance covered per week [km]	47.34 ± 16.10	49.00 ± 17.91

SD—standard deviation; cm—centimeters; kg—kilograms; km—kilometers.

**Table 2:** The muscle flexibility at baseline and after 6 weeks of exercising.

Outcome Measure		Group with FR (*n* = 32) Mean ± SD	*p* ^a^	Group without FR (*n* = 30) Mean ± SD	*p* ^a^	*p* ^b^
External rotation	Baseline	60.07 ± 9.21		59.53 ± 8.96		0.776
	After	60.77 ± 8.49	<0.001	60.06 ± 8.40	0.742	0.342
Iliopsoas L	Baseline	6.87 ± 3.74		6.93 ± 3.70		0.684
	After	5.45 ± 3.73	<0.001	5.48 ± 3.72	<0.001	0.120
Iliopsoas R	Baseline	6.60 ± 3.22		6.70 ± 3.12		0.599
	After	5.38 ± 3.55	<0.001	5.48 ± 3.49	0.001	0.178
Tensor fasciae latae L	Baseline	18.60 ± 9.17		17.87 ± 9.19		0.849
	After	21.03 ± 8.63	0.010	18.27 ± 8.76	0.331	0.598
Tensor fasciae latae R	Baseline	18.82 ± 9.11		17.75 ± 8.68		0.715
	After	21.53 ± 8.63	0.009	17.76 ± 8.11	0.964	0.851
Rectus femoris L	Baseline	27.02 ± 3.51		27.38 ± 3.20		0.833
	After	26.17 ± 3.28	<0.001	26.50 ± 2.91	0.192	0.608
Rectus femoris R	Baseline	26.92 ± 3.36		27.32 ± 3.09		0.801
	After	25.90 ± 3.04	<0.001	26.33 ± 2.80	0.296	0.432
Adductors	Baseline	73.03 ± 13.21		72.87 ± 13.35		0.848
	After	73.83 ± 13.37	0.061	73.77 ± 13.31	0.627	0.488

R—right side; L—left side; Baseline—the first measurement; After—measurement after application of self-myofascial release technique (in Group 1) and after 15 min without any intervention (in Group 2); SD—standard deviation; *p* ^a^—*p* value between first and second measurement within each group; *p* ^b^—*p* value between study groups. Values are expressed as Mean ± SD; bold—statistically significant.

## Data Availability

All data generated or analyzed during this study are included in this published article.
